# Molecular type distribution and fluconazole susceptibility of clinical *Cryptococcus gattii* isolates from South African laboratory-based surveillance, 2005–2013

**DOI:** 10.1371/journal.pntd.0010448

**Published:** 2022-06-29

**Authors:** Serisha D. Naicker, Carolina Firacative, Erika van Schalkwyk, Tsidiso G. Maphanga, Juan Monroy-Nieto, Jolene R. Bowers, David M. Engelthaler, Wieland Meyer, Nelesh P. Govender, for GERMS-SA

**Affiliations:** 1 National Institute for Communicable Diseases (Centre for Healthcare-Associated Infections, Antimicrobial Resistance and Mycoses), a Division of the National Health Laboratory Service, Johannesburg, South Africa; 2 School of Pathology, Faculty of Health Sciences, University of the Witwatersrand, Johannesburg, South Africa; 3 Studies in Translational Microbiology and Emerging Diseases (MICROS) Research Group, School of Medicine and Health Sciences, Universidad del Rosario, Bogota, Colombia; 4 Pathogen and Microbiome Division, Translational Genomics Research Institute, Flagstaff, Arizona, United States of America; 5 Molecular Mycology Research Laboratory, Centre for Infectious Diseases and Microbiology, Westmead Clinical School, Sydney Medical School, Faculty of Medicine and Health, The University of Sydney, Westmead, New South Wales, Australia; 6 Sydney Institute for Infectious Diseases, The University of Sydney, Westmead, New South Wales, Australia; 7 Westmead Institute for Medical Research, Westmead, New South Wales, Australia; 8 Research and Educational Network, Westmead Hospital, Western Sydney Local Health District, Westmead, New South Wales, Australia; 9 Curtin Medical School, Curtin University, Perth, Australia; 10 Division of Medical Microbiology, Faculty of Health Sciences, University of Cape Town, Cape Town, South Africa; 11 Medical Research Council Centre for Medical Mycology, College of Medicine and Health, University of Exeter, Exeter, United Kingdom; Rutgers University, UNITED STATES

## Abstract

As is the case globally, *Cryptococcus gattii* is a less frequent cause of cryptococcosis than *Cryptococcus neoformans* in South Africa. We performed multilocus sequence typing (MLST) and fluconazole susceptibility testing of 146 isolates randomly selected from 750 South African patients with *C*. *gattii* disease identified through enhanced laboratory surveillance, 2005 to 2013. The dominant molecular type was VGIV (101/146, 70%), followed by VGI (40/146, 27%), VGII (3/146, 2%) and VGIII (2/146, 1%). Among the 146 *C*. *gattii* isolates, 99 different sequence types (STs) were identified, with ST294 (14/146, 10%) and ST155 (10/146, 7%) being most commonly observed. The fluconazole MIC_50_ and MIC_90_ values of 105 (of 146) randomly selected *C*. *gattii* isolates were 4 μg/ml and 16 μg/ml, respectively. VGIV isolates had a lower MIC_50_ value compared to non-VGIV isolates, but these values were within one double-dilution of each other. HIV-seropositive patients had a ten-fold increased adjusted odds of a VGIV infection compared to HIV-seronegative patients, though with small numbers (99/136; 73% vs. 2/10; 20%), the confidence interval (CI) was wide (95% CI: 1.93–55.31, p = 0.006). Whole genome phylogeny of 98 isolates of South Africa’s most prevalent molecular type, VGIV, identified that this molecular type is highly diverse, with two interesting clusters of ten and six closely related isolates being identified, respectively. One of these clusters consisted only of patients from the Mpumalanga Province in South Africa, suggesting a similar environmental source. This study contributed new insights into the global population structure of this important human pathogen.

## Author summary

*Cryptococcus* is the most common cause of meningitis among adults in South Africa. Most human disease is caused by the members of two species complexes within the genus, *Cryptococcus neoformans* and *Cryptococcus gattii*. The environmental range of these species complexes, both found in soil, overlaps in southern Africa though *C*. *gattii* is a less common human pathogen. *C*. *gattii* is divided into six molecular types: VGI, VGII, VGIII, VGIV, VGV and VGVI. In earlier molecular epidemiology studies including relatively few isolates, most southern African isolates were confirmed as molecular type VGIV. We aimed to determine the molecular diversity of *C*. *gattii* in South Africa by genotyping patient isolates obtained through laboratory surveillance, 2005–2013. We confirmed that VGIV was the dominant molecular type and that HIV-seropositive patients were more likely to be infected with VGIV compared to those HIV-seronegative. Analysis of the genomes of South African VGIV isolates revealed that they spanned the whole VGIV clade and confirmed that most isolates did not cluster specifically. However, we observed two interesting clusters of closely related isolates, consisting of patients from three neighbouring provinces in South Africa, suggesting a similar environmental source. Further studies of clinical and environmental African *C*. *gattii* isolates are needed to gain a better understanding of this pathogen.

## Introduction

Basidiomycetous fungi within the genus *Cryptococcus* are the most common cause of meningitis among HIV-seropositive adults in southern Africa [1]. Human disease is mostly caused by two species complexes within the genus, *Cryptococcus neoformans* and *Cryptococcus gattii* [2], with *C*. *gattii* causing fewer than 1% of cases of cryptococcal disease globally [3]. While more often reported in the literature as causing disease in immunocompetent hosts without underlying medical conditions, *C*. *gattii* also causes disease in immunocompromised people [3–5]. People infected with *C*. *gattii* appear to present with pulmonary cryptococcosis more often than cryptococcal meningitis, with the opposite pattern being observed for disease caused by *C*. *neoformans* [4,6]. Among HIV-seropositive adults with advanced immunosuppression, *C*. *gattii* disease (meningitis with or without fungaemia) has been reported to be indistinguishable from disease caused by *C*. *neoformans* [6,7]. Regardless of the pathogen, flucytosine is recommended in combination with amphotericin B for induction treatment of HIV-associated cryptococcal meningitis, followed by fluconazole monotherapy for the consolidation and maintenance phases of treatment [8].

*C*. *gattii* was historically thought to be confined to tropical and subtropical regions and was found less frequently in temperate regions [9]. The environmental range of the two closely-related species overlaps in southern Africa, though *C*. *gattii* is a less common pathogen [10]. *C*. *gattii* is mainly associated with decomposing plant matter and certain tree species, such as eucalyptus in Australia and California [11,12], firs and oaks in the Pacific Northwest [13] and numerous tropical tree species (e.g. *Ficus* spp.) [14], with a new niche for *C*. *gattii* recently described after its isolation from hyrax faeces in an environmental study carried out in Zambia [12].

Since *C*. *gattii* is mainly restricted to sedentary trees, it is not as widely distributed as *C*. *neoformans*, which is hypothesized to have a global distribution owing partially to its ability to metabolise pigeon droppings [15]. *C*. *gattii* has also demonstrated the ability to adapt to new temperate niches, causing a geographically restricted outbreak in North America [13,15–17]. There are many theories on the unexpected and unexplained emergence of *C*. *gattii* in North America. A recently-published hypothesis suggests that *C*. *gattii* was transported by ships in contaminated ballast water tanks from South America to North America in 1914 [18]. This organism then established itself in coastal waters. A tsunami in 1964 may then have carried *C*. *gattii* into the coastal forests. It adapted subsequently to a new environmental niche (i.e. land/forest) causing human infections about three decades later [19]. While *C*. *neoformans* has been hypothesized to originate from Africa, *C*. *gattii* could have originated from South America considering the high genetic diversity of South American *C*. *gattii* isolates [10,20].

*C*. *gattii* is divided into six molecular types: VGI, VGII, VGIII, VGIV, VGV and VGVI [21,22], of which five have been proposed as separate species [23], with the molecular type VGV recently identified from six environmental isolates collected from the Zambian Central Miombo woodlands [22]. VGI is mostly found in Australia and Papua New Guinea [24]. VGII is prevalent in Canada and the United States of America (USA), where it has caused a major outbreak [13]. This molecular type is also widely present in Australia [25], Brazil and Colombia [26]. VGIII has been found in the USA [27], Mexico, Brazil and Colombia [26]. The molecular type VGIV has until now been mainly identified from India [28], Colombia, Mexico [29], Botswana, Malawi [30] and Zimbabwe [31]. VGVI has been newly described based on two identical isolates from Mexico [23]. In earlier molecular epidemiology studies including relatively few isolates, most southern African isolates were confirmed as molecular type VGIV [30,32], with this molecular type being mostly associated with disease in HIV-infected individuals [4].

To expand on these earlier findings, we aimed to: (1) determine the molecular diversity of *C*. *gattii* in South Africa by genotyping a subset of clinical isolates from enhanced laboratory-based surveillance conducted between 2005 and 2013; (2) describe the fluconazole susceptibility of these isolates, and (3) determine if there was an association between patient clinical characteristics and the infecting molecular type, and the effect of molecular type on in-hospital mortality. In addition, we analysed the genomes of 98 isolates of the most common South African *C*. *gattii* molecular type VGIV to place them into a global context.

## Materials and methods

### Ethics statement

Ethics clearance for this study was obtained from the Human Research Ethics Committee (Medical), University of the Witwatersrand with clearance certificate numbers: M160375 and M1809107. All data analysed was anonymized.

### Study design and sample selection

We conducted a cross-sectional study nested within laboratory-based surveillance for cryptococcosis in South Africa from 2005 to 2013. We used this time period since routine collection and storage of cryptococcal isolates through surveillance was terminated in 2013. From 1 January 2005 to 30 June 2008, cryptococcal isolates were submitted from all laboratories to the National Institute for Communicable Diseases (NICD). The surveillance methodology changed from 1 July 2008 to 31 December 2013 when only enhanced surveillance sites (ESS) (29 hospitals in 9 provinces), National Health Laboratory Service (NHLS) laboratories in KwaZulu-Natal Province, and pathology laboratories in the private, mining, and military sectors were required to submit isolates. A case was defined as a person diagnosed with cryptococcal disease by any one of the following positive tests during a 30-day period: (1) India ink microscopy on cerebrospinal fluid (CSF) or (2) a positive cryptococcal antigen test on blood or CSF or (3) culture of *Cryptococcus* from any clinical specimen. Recurrent isolates from the same patients were excluded and only cases with cultured and viable isolates were included [33]. We also excluded isolates that were identified as *C*. *neoformans* or other *Cryptococcus* species. Following these inclusion and exclusion criteria, 750 cases of *C*. *gattii* infection in total were available for analysis. HIV infection status was recorded for 387 of these patients at ESS; we excluded all other patients with an unknown HIV infection status. Among these 387 patients, 374 were HIV-seropositive and 13 were HIV-seronegative. One hundred and thirty-six *C*. *gattii* isolates were randomly selected from the 374 HIV-seropositive cases using a random-integer generator (https://www.random.org/integers/) and all 13 isolates from HIV-seronegative persons were selected but only ten were viable. In total, 146 unique cases were selected from 387 cases of *C*. *gattii* disease between 2005 and 2013 and the corresponding isolates were genotyped (S1 Fig).

### Subculture and identification of surveillance isolates

Following primary isolation of *Cryptococcus* species at diagnostic pathology laboratories, a sweep of the culture plate was inoculated onto Dorset medium (Diagnostic Media Products (DMP), NHLS, Johannesburg, South Africa) and transported to a reference laboratory at the NICD in Johannesburg, where the isolates were identified by phenotypic methods [34] and stored at -70°C. The patient metadata that accompanied each isolate were captured in a surveillance database. The selected *C*. *gattii* isolates were retrieved from -70°C storage and sub-cultured onto Sabouraud dextrose agar (DMP, NHLS, Johannesburg, South Africa) to check for purity and viability [33]. We phenotypically characterised isolates as previously described [34].

### Multilocus sequence typing (MLST) and data analysis

DNA was extracted from single yeast colonies using the Zymo ZR Fungal/Bacterial DNA MiniPrep kit (Zymo Research Corp, USA) following the manufacturer’s instructions. The only modification from the manufacturer’s instructions was the starting material for DNA extractions, since single yeast colonies grown on Sabouraud agar (DMP, NHLS, Johannesburg, South Africa) at 30°C after 48h were used. The International Society for Human and Animal Mycology (ISHAM) MLST consensus scheme for *C*. *neoformans* and *C*. *gattii* containing six housekeeping genes: *CAP59*, *GPD1*, *LAC1*, *PLB1*, *SOD1*, *URA5* and the intergenic spacer region IGS1 was used as previously published [21] for genotyping. DNA was amplified using a conventional PCR with DreamTaq Hot Start DNA Polymerase (Thermo Fisher Scientific, Waltham, Masschusetts, USA) in an Applied Biosystems 2720 Thermal cycler (Thermo Fisher Scientific, Waltham, Masschusetts, USA) using PCR cycling conditions described previously [21]. Amplicons were visualised on a 1% agarose gel and Sanger sequencing was performed. For each locus, DNA sequences in both forward and reverse directions were obtained and edited using the program Sequencher ver. 5.4.6 (http://www.genecodes.com). Allele types (ATs) and sequence types (STs) were assigned using the online *C*. *gattii* MLST database (http://mlst.mycologylab.org). The concatenated DNA sequences for the seven loci (S1 Table) were aligned using the program ClustalW (BioEdit Sequence Alignment Editor). A phylogenetic tree was generated with the program MEGA 5 using the neighbour-joining method with a bootstrap analysis of 100 replicates and the Jukes-Cantor model [35].

Mating type identification was performed by conventional PCR amplification using primer sets and cycling conditions from previous studies [36,37], which are specific to the mating type regions of a and α mating-type cells. Amplicons were visualised on a 1% agarose gel and the mating type was determined using amplification that related to each mating-type’s expected amplicon size [36,37].

### Fluconazole susceptibility testing

Of the 146 *C*. *gattii* isolates, fluconazole susceptibility testing was performed for 105 isolates: 60 randomly selected VGIV (from a total of 101), 40 VGI, three VGII and two VGIII. We determined the fluconazole minimum inhibitory concentration (MIC) values (range: 0.125 μg/ml to 64 μg/ml) using custom-made broth microdilution (BMD) panels (NICD, Johannesburg) prepared, inoculated and read according to susceptibility testing methods published previously and the Clinical and Laboratory Standards Institute (CLSI) M27-A3 and M60 recommendations [38–40]. We interpreted fluconazole MIC values using published epidemiological cut-off values (ECVs) for the VGIII and VGIV isolates [41], and the CLSI ECVs for the VGI and VGII isolates [42].

### Whole genome sequencing and phylogenetic analysis

Whole genome sequencing (WGS) was performed on 98 molecular type VGIV isolates using Illumina MiSeq sequencing technology (Illumina, San Diego, California, USA). Previously extracted DNA samples (extraction procedure described above) were prepared for paired-end sequencing using the NEBNext Ultra II DNA Library Prep Kit for Illumina followed by 2 × 300 bp sequencing on a MiSeq instrument at the Translational Genomics Research Institute (Pathogen and Microbiome Division, Flagstaff, USA). Single nucleotide polymorphisms (SNPs) were detected from raw read data using the publically available Northern Arizona SNP pipeline (NASP) [43]. The pipeline was set to align reads for every sample to the known assembled reference VGIV genome (IND 107, NCBI BioSample number: SAMN01932842) using Burrows-Wheeler Aligner (BWA) [44]. SNP variants were detected using SAMtools [45]. Posterior filtering parameters involved removing positions that had <10x coverage, <90% variant allele calls, and those mapping to duplicated regions in the reference. Downstream filtering by NASP produced the final high-quality or BestSNP alignment that was then used for maximum parsimony inference. Only positions present in all genomes with at least 10x depth of coverage and 90% agreement were included. Maximum parsimony trees with 100 bootstrap replicates were constructed using the phangorn library [46] and visualized using MEGA 6 software [47]. We included 47 additional global VGIV whole genome sequences. Nineteen VGIV isolates were obtained from the Australian Medical Mycology Culture Collection (WFCC registration number: WM-1205) and sequenced herein (S2 Table). The remaining VGIV whole genome sequences were obtained from NCBI GenBank; six of which were sequenced in previous studies [17,22,48]. The 47 additional VGIV genomes included 19 isolates from Botswana, 17 isolates from South Africa, four isolates from Colombia, three isolates from India, two isolates from Australia, one isolate from Zambia and the geographical origin of one isolate was unknown. For assessment of specific clusters, trees were drawn for each obtained cluster separately to maximize the high-resolution of comparable positions between samples of each apparent clade. For these specific sub-analyses, draft assemblies of one of the samples in each clade were created *de*-*novo* using SPAdes (https://cab.spbu.ru/software/spades/) in careful mode and then used as references for the cluster trees. A cluster was defined as a group of more than two isolates that shared a common ancestor with fewer than 1100 SNPs between isolates. All WGS data were deposited in the NCBI SRA under the BioProject accession number PRJNA804139.

### Statistical data analysis

For the 2005–2013 surveillance period, we compared demographic characteristics of patients with *C*. *gattii* and *C*. *neoformans* disease to determine if there were any differences between these two species complexes. For *C*. *gattii* infection, we used logistic regression models to determine associations between patient characteristics and molecular type. Patient case data were collected by nurse surveillance officers at ESS who either interviewed patients or reviewed their medical charts [33]. We defined the molecular type groups as VGIV vs. non-VGIV, since VGIV was the dominant molecular type in South Africa. We included the following variables in the analysis with molecular type as the dependent variable: sex, age, year of diagnosis, geographical region, specimen type, HIV infection status, mental status at diagnosis, CD4+ T-cell (CD4) count at diagnosis, antiretroviral treatment, current antifungal treatment, and tuberculosis treatment. Mental status was categorised as “alert” (a Glasgow Coma Scale [GCS] score of 15) or “not alert” (a GCS score <15 or a patient was recorded to be disorientated, stuporose or comatose at the time of diagnosis) [33]. We divided the geographical regions of South Africa, based on the Koppen-Gieger climate classification zones [49], into mostly temperate (Gauteng, Mpumalanga, KwaZulu-Natal and Western Cape provinces) vs. mostly arid (Northern Cape, Free State, Limpopo, North West and Eastern Cape provinces). In the final multivariable analysis, we only adjusted for factors that were associated with geographical region and were risk factors for infection caused by VGIV isolates; the variables age, sex and HIV infection status all met these criteria. We also separately modelled the effect of molecular type on in-hospital mortality, adjusting for sex, age, mental status at diagnosis, CD4 count at diagnosis, antiretroviral treatment, and current antifungal treatment. We compared the fluconazole MIC_50_ values of the VGIV isolates and non-VGIV isolates using a Wilcoxon rank-sum test. We also compared the association between MIC value (≤8 μg/ml vs >8 μg/ml) and outcome data of patients infected with *C*. *gattii*. Statistical analyses were performed using Stata statistical software ver. 14.1 (StataCorp LP, Texas, USA).

## Results

### Descriptive analysis of laboratory-based surveillance

From 2005 to 2013, 25,676 viable *Cryptococcus* isolates were collected from 24,286 cases of cryptococcal disease. Only 3% of the viable isolates (781/25,676) were identified as *C*. *gattii* originating from 750 patients compared to 97% (24,871/25,676) of viable *C*. *neoformans* isolates originating from 23,512 patients. Owing in part to changes in surveillance methodology, the number of culture-confirmed *C*. *gattii* cases varied by year: 2005: 114 (15% of total), 2006: 133 (18%), 2007: 91 (12%), 2008: 61 (8%), 2009: 75 (10%), 2010: 65 (9%), 2011: 58 (8%), 2012: 56 (7%) and 2013: 97 (13%). The median age for patients infected with *C*. *gattii* or *C*. *neoformans* was 35 years, though the inter-quartile ranges (IQR) differed slightly (*C*. *gattii*: IQR, 30–42 years vs. *C*. *neoformans*: IQR, 29–41 years). In terms of gender differences of cases infected by the two species complexes, 57% (426/748) of patients infected with *C*. *gattii* were male while 48% (11,057/23,206) infected with *C*. *neoformans* were male (p = 0.0001). More patients diagnosed with either *C*. *neoformans* or *C*. *gattii* disease were from the mostly temperate provinces of South Africa (*C*. *gattii*: 565/750, 75%; *C*. *neoformans*: 17,682/23,512, 75%) compared to the mostly arid provinces (*C*. *gattii*: 185/750, 25%; *C*. *neoformans*: 5,830/23,512, 25%). For both *C*. *gattii* and *C*. *neoformans* infections, CSF was the most common specimen type from which the pathogen was cultured (*C*. *gattii*: 712/750, 95%; *C*. *neoformans*: 22,279/23,512, 95%) followed by blood culture (*C*. *gattii*: 32/750, 4%; *C*. *neoformans*: 1179/23,512, 5%).

### Genotyping of 146 clinical *C*. *gattii* isolates

**Fig 1:**
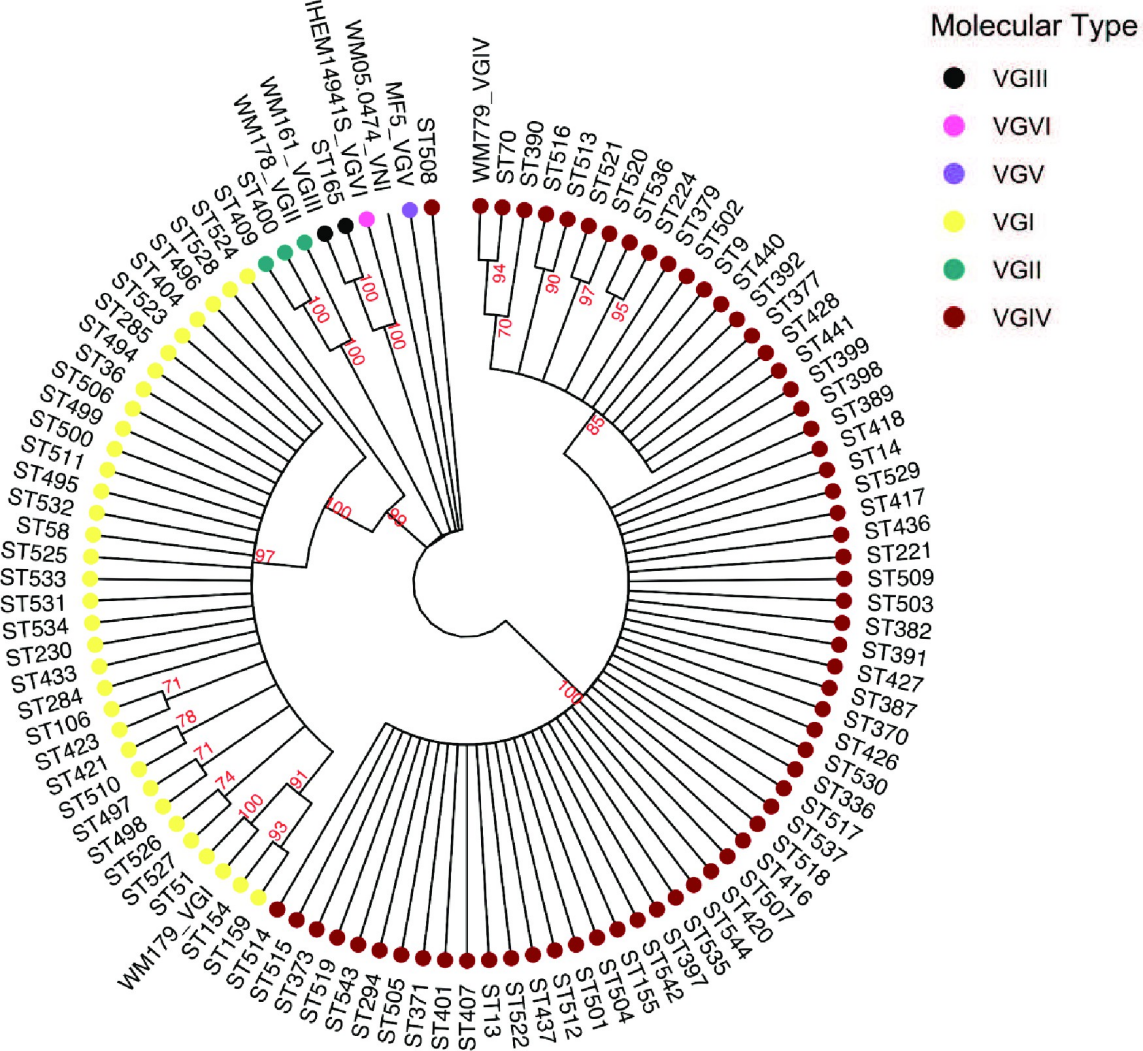
Neighbour joining phylogenetic tree based on multilocus sequence typing data showing clustering of the molecular types for 99 different sequence types (STs) from 146 South African clinical *Cryptococcus gattii* isolates. *Cryptococcus neoformans* strain WM 05.474, molecular type VNI serves as the outgroup. A standard strain for each molecular type was also included. Bootstrap analysis was performed with 100 bootstrap replicates. Bootstrap values are shown next to the branches.

We genotyped 146 isolates from a total of 387 cases between 2005 and 2013, equalling 38% of all cases from ESS. The molecular type distribution of 146 *C*. *gattii* isolates (S1 Supporting Information) was as follows: VGIV (101/146, 70%; 95% CI: 0.61–0.77) followed by VGI (40/146, 27%; 95% CI: 0.20–0.35), VGII (3/146, 2%; 95% CI: 0.004–0.06) and VGIII (2/146, 1%; 95% CI: 0.002–0.05). Most isolates had the MATα mating type (n = 139, 95%), whereas 5% (n = 7, 5 VGIV and 2 VGI) were MATa. There were 99 different STs observed amongst the 146 *C*. *gattii* isolates (Fig 1). ST294 (13/146, 9%) and ST155 (10/146, 7%) were the most commonly observed STs.

### Association between patients’ clinical characteristics and molecular type

After adjusting for sex, age and HIV infection status, patients diagnosed in the mostly temperate provinces of South Africa had a 43% reduced odds of a VGIV infection than patients from mostly arid provinces, though the 95% CI spanned 1 (0.21–1.58) and the p-value was large (0.28) (S3 and S4 Tables). We also found that HIV-seropositive patients had a ten times increased odds of a VGIV infection compared to those who were HIV-seronegative in the same adjusted analysis, though with small case numbers, the CI was very wide (95% CI: 1.93–55.31, p = 0.006) (S3 and S4 Tables).

### Association between molecular type and in-hospital mortality

The overall crude in-hospital case-fatality ratio was 28% (40/142). The case-fatality ratio was 30% (29/98) for patients infected with *C*. *gattii* isolates of the VGIV molecular type versus 25% (11/44) for patients infected with a non-VGIV molecular type (crude odds ratio: 1.26, 95% CI: 0.56–2.84, p = 0.57) (S5 Table). On multivariable analysis, patients infected with the VGIV molecular type had a 43% reduced adjusted odds of dying than those infected with other molecular types, though the 95% CI crossed 1 (0.07–4.43) and the p-value was large (0.59) (S6 Table).

### Fluconazole susceptibility testing

The average fluconazole MIC_50_ and MIC_90_ values for all 105 tested *C*. *gattii* isolates (60 VGIV, 40 VGI, 3 VGII and 2 VGIII) were 4 μg/ml and 16 μg/ml, respectively, with a geometric mean of 4.53 μg/ml (Table 1). There was a statistical difference in the MIC_50_ values between the VGIV isolates and non-VGIV isolates (p = 0.001), though these differed by only one double-dilution (4 μg/ml for VGIV isolates vs. 8 μg/ml for non-VGIV isolates) (Table 1). Most of the VGI, VGII and VGIV isolates had a MIC value of ≤8 μg/ml, which is considered to be wild-type according to published ECVs [41,42]. However, the two VGIII isolates, six VGIV isolates and six VGI isolates were considered to be non-wild-type [41,42]. On unadjusted analysis, patients with a cryptococcal isolate that had a fluconazole MIC value of greater than 8 μg/ml were more than one and a half times more likely to die, though this estimate also had a 95% CI spanning 1 and a large p-value (95% CI: 0.47–5.64; p = 0.43).

**Table 1:** The fluconazole minimum inhibitory concentration (MIC) distribution, MIC_50_, MIC_90_, geometric mean and range (in μg/ml) represented by molecular type of the 105 tested South African *Cryptococcus gattii* isolates from enhanced laboratory-based surveillance collected between 2005 and 2013.

Molecular Type	Total	MIC Value (μg/ml)							MIC_50_ (μg/ml)	MIC_90_ (μg/ml)	Geometric Mean	Range
		0.5	1	2	4	8	16	32				
VGI	40	0	1	5	9	19	5	1	8	16	6.17	1–32
VGII	3	0	0	0	0	3	0	0	8	8	8	8
VGIII	2	0	0	0	0	2	0	0	8	8	8	8
VGIV	60	3	7	14	17	13	5	1	4	12	3.52	0.5–32
**Total**	**105**	**3**	**8**	**19**	**26**	**37**	**10**	**2**	**4**	**16**	**4.53**	**0.5–32**

### Whole genome sequencing of 98 *C*. *gattii* VGIV isolates

**Fig 2:**
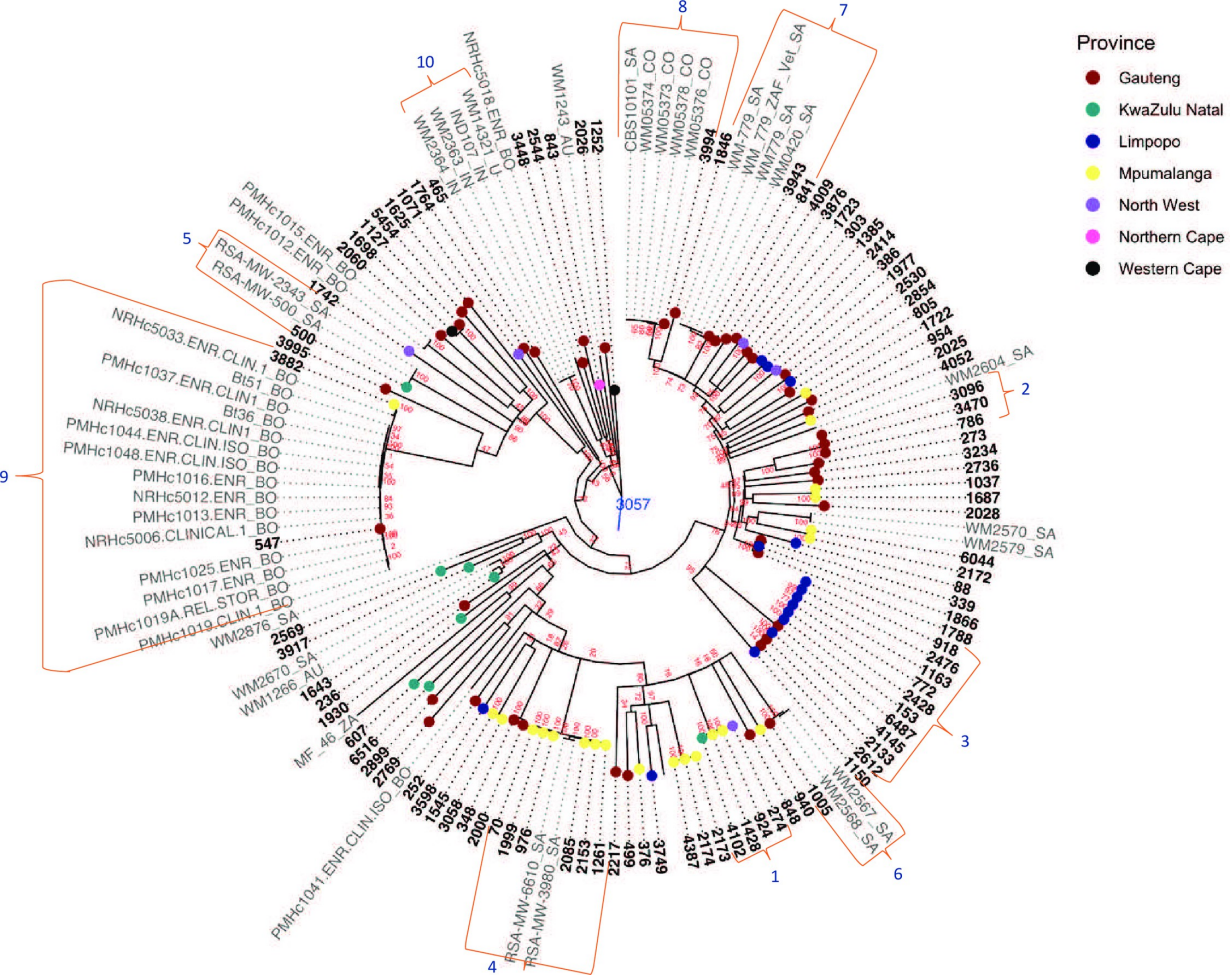
Rooted maximum parsimony tree generated from the WGS SNP analysis of 98 South African clinical VGIV *Cryptococcus gattii* isolates (coloured circles and bolded tip labels) and 47 global samples. IND107 was used as the reference. BWA was used to align reads to the reference and SAMtools was used to call SNPs. There were 123,891 SNPs in total with a coverage breadth of 61%. Bootstrap analysis was peformed with 100 bootstrap replicates. The bootstrap values are shown next to the branches. The consistency index was 0.35 and the number of phylogenetic informative positions were 123,891. BO–Botswana, SA–South Africa, CO–Colombia, AU–Australia, IN–India, ZA–Zambia, U—Unknown.

In order to place our South African VGIV isolates into a global context, we compared the WGS data between South African VGIV isolates and global VGIV isolates. Fig 2 shows the WGS SNP analysis of 98 VGIV isolates from this study, the VGIV reference strain IND 107 and 47 global VGIV isolates. There were 123,891 SNPs called. The overall minimum, median and maximum number of SNPs among all 146 isolates was 0; 13,309 and 21,804, respectively. Kmer-based typing of these genomes *in-silico* using a custom kraken database indicated that none of our isolates belonged genetically to the new molecular type VGV identified recently in Zambia [22]. Scattered throughout the tree, there were ten clusters overall (see labelled clusters in Fig 2 and Table 2). Seven clusters included South African VGIV isolates only. Cluster 1 consisted of four isolates. The median number of SNPs was 198 between three isolates (924, 1428 and 274). The fourth isolate (4102) was separated by 850 SNPs from the other three isolates in this cluster. Isolate numbers 924 and 1428 were from patients living in the same town, in the Mpumalanga Province, diagnosed at the same facility just over two months apart in 2011. Isolates 3096, 786 and 3470 were in cluster 2 and the median number of SNPs was 404. All three patients from whom these isolates originated lived in the Gauteng Province and two patients were diagnosed in the same facility less than two months apart in 2013. There were two interesting clusters of closely related isolates observed (clusters 3 and 4, Fig 2 and Table 2). Cluster 3 consisted of ten isolates from patients living in the Gauteng and Limpopo provinces named the Gauteng/Limpopo cluster and cluster 4 consisted of six isolates from patients living in the Mpumalanga Province named the Mpumalanga cluster. The Mpumalanga cluster also contained two isolates from a previously published South African study [48]. There were fewer than 30 SNPs between two previously published sequences of South African isolates cultured from a South African patient with recurrent cryptococcosis between 2005 and 2009 [48] and South African isolate number 500 from our study cultured from a patient living in the KwaZulu-Natal Province diagnosed in January 2007 (cluster 5, Fig 2 and Table 2). There were fewer than 700 SNPs between isolate number 1005 and two South African samples sequenced from the Australian Medical Mycology Culture Collection in cluster 6. In cluster 7, there were two isolates from our study (numbers 3943 and 841) collected in 2008 and 2011 respectively, obtained from patients living in Gauteng. This cluster also included South African samples (WM0420 and WM779) sequenced from the Australian Medical Mycology Culture Collection separated from each other by less than 20 SNPs but separated from isolate number 841 and isolate number 3943 by fewer than 1100 SNPs.

**Table 2:** Description of clusters of *Cryptococcus gattii* VGIV isolates from the WGS SNP analysis shown in Fig 2.

Cluster number	Number of isolates	Minimum number of SNPs	Median number of SNPs	Maximum number of SNPs	Patients’ locations	Years of isolation
1	4	0	305	844	KwaZulu Natal, North West and Mpumalanga	2008, 2009 and 2011
2	3	0	404	652	Gauteng	2011 and 2013
3	10	52	426	574	Gauteng and Limpopo	2006, 2008, 2009, 2010, 2011 and 2013
4	8	0	598	699	Mpumalanga	2006, 2008, 2009, 2010 and 2011
5	3	0	8	30	KwaZulu Natal	2005–2009 and 2007
6	3	0	2	694	Gauteng	1996 and 2007
7	4	0	872	1064	Gauteng	1994, 1996, 2008 and 2011
8	6	0	6	478	Gauteng	2008
9	17	0	318	908	Gauteng and Mpumalanga	2000, 2009, 2012 and 2013
10	4	0	3	50	India	1997

There were two clusters that included South African VGIV isolates from our study and some of the VGIV isolates from other countries. The minimum, median and maximum number of SNPs were 0, 6 and 478 SNPs, respectively, between four Colombian isolates [50], a publically available sequence from a South African animal isolate and isolate number 3994 (cluster 8, Fig 2 and Table 2). The patient infected with isolate number 3994 was from the Gauteng Province and was diagnosed in September 2008. The minimum, median and maximum number of SNPs were 0, 318 and 908 SNPs, respectively, between isolate number 547 (patient from Gauteng Province) and isolate number 3882 (patient from Mpumalanga Province) and fifteen Botswanan samples that were isolated from CSF in 2000 and 2012 (cluster 9, Fig 2 and Table 2). There was one cluster that contained no South African VGIV isolates (cluster 10, Fig 2). This cluster included two Indian isolates (isolate numbers: WM2363 and WM2364), an isolate with unknown origin (WM14321) as well as the reference VGIV isolate (IND107). There were less than five SNPs between isolates WM2363, WM2364 and WM14321 with at most 50 SNPs between these three isolates and the reference.

**Fig 3:**
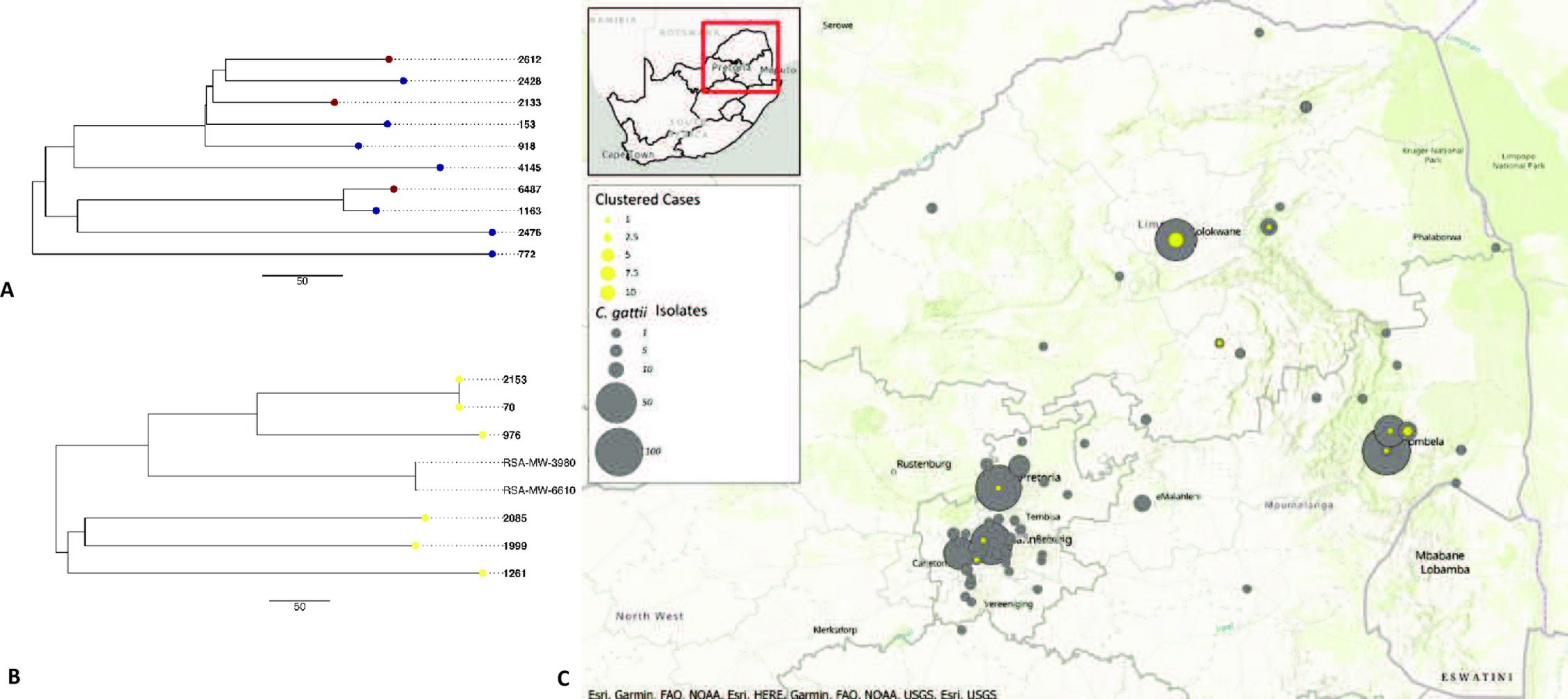
All clinical cases of *Cryptococcus gattii* infection (n = 508) collected through laboratory-based surveillance during 2005–2013 from three South African provinces highlighting the cases that clustered closely by genomic data. A: Unrooted maximum parsimony tree generated from the WGS SNP analysis of ten South African clinical VGIV *C*. *gattii* isolates from patients living in the Gauteng and Limpopo provinces. Strain number 918 was used as the reference. There were 1600 SNPs in total with a coverage breadth of 96%. The consistency index was 0.99 and the number of phylogenetic sites were 1600. Maroon–Gauteng, purple–Limpopo. B: Unrooted maximum parsimony tree generated from the WGS SNP analysis of eight South African clinical VGIV *C*. *gattii* isolates (six from this study and two from a previously-published South African study [48]). Isolates from this study are indicated in bold. Strain number 976 was used as the reference. There were 1649 SNPs in total with a coverage breadth of 96%. The consistency index was 1.00 and the number of phylogenetic sites were 1649. Yellow–Mpumalanga Province. C: Map of South Africa showing the clustered cases in yellow and all *C*. *gattii* cases (n = 508) in grey from Gauteng, Limpopo and Mpumalanga provinces (https://cdn.arcgis.com/sharing/rest/content/items/7dc6cea0b1764a1f9af2e679f642f0f5/resources/styles/root.json). This map was created using ArcGIS software by ESRI (www.esri.com). ArcGIS and ArcMap are the intellectual property of Esri and are used herein under license. Copyright Esri. All rights reserved. For more information about Esri software, please visit www.esri.com.

We then analysed the Gauteng/Limpopo cluster and Mpumalanga cluster separately using the draft assembly of one of our isolates (isolate numbers 918 and 976, respectively) as a reference in NASP. There were 1600 SNPs called within the Gauteng/Limpopo cluster of ten isolates (Fig 3A and 3C). The minimum, median and maximum number of SNPs were 52, 426 and 574 SNPs, respectively. Six patients from whom these isolates originated were male and four patients were female. Seven patients were from the Limpopo Province; four of which were diagnosed at the same facility; and three were from the Gauteng Province. The cases did not cluster in time: one patient each was diagnosed in December 2006 and February 2008, three patients each in January, November and December of 2009, one patient each in May 2010 and August 2011, and one patient each in January, April and November 2013 (S7 Table). The map in Fig 3C highlights the clustered cases in yellow from the Gauteng, Limpopo and Mpumalanga Provinces. The ten patients in the Gauteng/Limpopo cluster were all located in towns within a 355 km radius. The Gauteng patients were from three towns located within a 70 km radius. The Limpopo patients were from two towns also located within a 70 km radius.

The Mpumalanga cluster of six isolates from our study and two from a previously published South African study [48] contained 1649 SNPs among all genomes (Fig 3B and 3C). The minimum, median and maximum number of SNPs were 0, 598 and 699 SNPs, respectively. The six isolates from our study originated from five male patients and one female patient, with five patients diagnosed at the same facility. The cases did not cluster in time: one patient each was diagnosed in May 2006, April 2008, June 2009, January 2010, December 2010 and July 2011 (S8 Table). The two previously published sequences were from isolates cultured from another South African patient with recurrent cryptococcosis between 2005 and 2009 [48]. Two isolates, number 70 and number 2153, within this cluster were distanced by zero SNPs between them. These patients lived in two towns that were about 30 kilometres apart, but both were diagnosed at the same facility two years apart (2008 and 2010) (S8 Table). As mentioned before, the map in Fig 3C highlights the clustered cases from the Mpumalanga, Gauteng and Limpopo Provinces shown in yellow. The patients from the Mpumalanga Province with isolates that were closely related were all located in four towns in close proximity (within a radius of 51 km).

## Discussion

Laboratory-based surveillance for cryptococcosis, a common HIV-associated opportunistic infection in South Africa, provided a platform to determine the molecular epidemiology of *C*. *gattii*. Only 3% of viable isolates were identified as belonging to the *C*. *gattii* species complex with the remainder identified as belonging to the *C*. *neoformans* species complex. Previous clinical studies from neighbouring southern African countries have reported a much higher proportion of *C*. *gattii* infections, such as 13% in Botswana and Malawi [30] or 17% in Zimbabwe [31]. However, these studies included small case numbers, where cryptococcal isolates were obtained from patients admitted to a single facility or cases were not randomly selected from a defined study population.

We observed 99 STs among our clinical *C*. *gattii* isolates, indicating a high genetic diversity, with VGIV being the dominant molecular type in South Africa. This confirms the results of studies undertaken in Zimbabwe, Botswana and Malawi [30, 31]. In an earlier South African study on isolates from episodes of recurrent cryptococcosis, there were eight *C*. *gattii* isolates recovered from four patients and four isolates each had the molecular type VGI and VGIV, respectively [34]. In our larger study, we were able to describe the molecular type distribution of *C*. *gattii* in South African patients. Twenty-seven per cent of the clinical isolates in our study had the VGI molecular type; this molecular type has been described in a previous study as being dominant among Kenyan clinical and environmental isolates [51]. In a study of 350 clinical, veterinary and environmental *C*. *gattii* isolates from Africa, Asia, Europe, North America and South America, VGII was the dominant molecular type followed by VGI [52]. There were lower percentages of VGIII and VGIV isolates. For the twenty-eight African isolates (countries unspecified), accounting for 8% of the total number of isolates included in this global study, VGI was the dominant molecular type, followed by VGIV. The authors concluded that VGI and VGII are globally distributed whereas VGIII and VGIV are minor molecular types that seem to be geographically restricted to specific locations [52]. However, in this specific study, a larger number of isolates was obtained from Europe, North America and South America, and therefore this collection was not representative of the global molecular type distribution.

The fluconazole MIC_50_ values, MIC_90_ values and ranges overlapped for isolates with all four molecular types in our single-centre study. Our study therefore does not provide sufficient evidence for any meaningful difference in fluconazole susceptibility in relation to molecular type. Compared to the fluconazole MIC data for *C*. *neoformans* isolates from a previous South African study using the same surveillance platform, the MIC_50_ and MIC_90_ values for *C*. *gattii* differed by two fold higher dilutions [33]. A previous study showed that there was a higher level of heteroresistance to fluconazole in *C*. *gattii* than in *C*. *neoformans* [53]. These *C*. *gattii* isolates were also more resistant to xenobiotics than *C*. *neoformans* which could be due to the ecological niche *C*. *gattii* inhabits since tree hollows may harbour various ubiquitous saprophytic microorganisms that produce xenobiotics [53]. All *C*. *gattii* isolates showed heteroresistance in this one study; therefore, this is an intrinsic property of *C*. *gattii* that the authors speculate reflect an acquisition from its ecological niche but this needs to be studied further [53].

We found incidentally that HIV-seropositive patients had an increased odds of a VGIV infection compared to those HIV-seronegative, after adjusting for age, sex and climatic region. With a small sample size, our estimate of this effect is likely to be imprecise. However, previous studies in southern Africa have also shown that the VGIV molecular type is a major cause of *C*. *gattii* meningitis in HIV-seropositive patients [30,31]. In general, VGIV and VGIII have been reported to cause infection in HIV-seropositive patients whereas VGI and VGII are reported to cause infection mainly in immunocompetent hosts [4,5,54,55]. We are uncertain of what could explain this difference but Harris and colleagues previously speculated that the *C*. *gattii* molecular type, sequence type, fungal population genetics and environmental distribution “probably interact to mediate infection in patients with varying degrees of immune competence” [56]. Based on the point estimate, patients infected with the VGIV molecular type were less likely to die in hospital compared to patients infected with the non-VGIV molecular types, although we may have found this difference entirely by chance with a large p-value. Beale et al. observed the opposite for *C*. *neoformans* whereby the VNB genotype was associated with a worse survival outcome [57]. Recent phenotypic variation results suggest that the VGIV molecular type may be a less virulent pathogen [58]. These authors observed that the VGIV molecular type is similar to other molecular types in terms of capsule production and cell size. Both VGIV and VGIII molecular types were more sensitive to temperature compared to VGI and VGII that are considered most virulent [58]. VGIV also produces irregular cells, a variant phenotype not shown to be related to patient death when compared to other variant phenotypes, such as giant cells, micro cells and shed extracellular capsule produced by *Cryptococcus* species [58]. There is also evidence that virulence is not specifically associated with a certain molecular type [59,60]. In one of these studies, *Galleria mellonella* larvae were infected with the four *C*. *gattii* molecular types and highly virulent isolates were observed from all molecular types, suggesting that virulence may be related to strain-specific characteristics regardless of the molecular type [59].

In our WGS analysis, we observed a total of ten closely-related clusters and we zoomed in on two clusters specifically which we called the Gauteng/Limpopo cluster and Mpumalanga cluster consisting of ten and six isolates, respectively. The median number of SNPs for each of these two clusters were 426 and 598, respectively. A previous study described nine VGI isolates isolated from patients living in the south-eastern states of the USA that clustered together with 41,000 SNPs and 4558 SNPs between any two isolates [61]. Firacative and colleagues were able to define two major clusters of VGIII isolates in their study, separating serotype B from serotype C isolates. There were 88,337 SNPs for one cluster and 79,945 SNPs for the second cluster [60]. Three clusters were observed amongst the VGII outbreak isolates in the Pacific North West region of North America, made up of VGIIa, VGIIb and VGIIc, with 107, 132 and 137 SNPs within each of those subtypes, respectively, indicating that these VGII populations in that region were clonal with a low genetic diversity [17,61]. Our results suggest that, like VGI and VGIII, the VGIV molecular type also has a high genetic diversity. We observed that one of the isolates from our study and a previously isolated South African sample were related to four previously isolated environmental Colombian VGIV isolates with <480 SNPs [50]. This suggests some level of relatedness between VGIV isolates from different geographical regions or a similar environmental exposure, although we do not have the travel history of the patients to confirm this. The possible relatedness between Colombian and South African isolates could be due to genetic exchange between *C*. *gattii* molecular types before the continental drift [20,62]. According to one hypothesis, before the ancient supercontinent Pangea split into the present continents, *C*. *neoformans* and *C*. *gattii* shared a common Pangean ancestor somewhere in between South America and Africa since these two continents were next to each other on Pangea [20]. This continental drift dispersal hypothesis has further been applied to the molecular types within the *C*. *gattii* species complex [62].

Our surveillance data also consisted of the patients’ residential addresses and healthcare facility at the time of clinical diagnosis. However, this does not take into account the long latency period of cryptococcosis in some cases and relocation of residence between provinces. The Gauteng, Limpopo, North West and Mpumalanga Provinces are located in the northern parts of South Africa. These provinces are inland. However, climatic differences between these provinces do exist. The Gauteng Province is elevated and temperate, whereas parts of the Mpumalanga Province are low-lying, subtropical to tropical and humid but also dry in some areas. The North West Province and the Limpopo Province are hot and dry. There was a cluster of six isolates from patients living in the Lowveld region of Mpumalanga Province in South Africa, suggesting a common environmental source. Based on our findings, we can speculate that the VGIV molecular type may be widely distributed in this subtropical environment. The reason for its widespread environmental distribution could be due to this genotype’s high fitness in the environment. This has been observed with triazole-resistant *Aspergillus fumigatus* with the TR/L98H substitution [63] as well as in *C*. *gattii* in a recent study done in Zambia [22]. In the Zambian study, *C*. *gattii* was mostly associated with the Zambian Central Miombo woodlands that is dry and tropical [12]. The genomes of six of these isolates were recently sequenced and found to represent a new molecular type, VGV [22]. This molecular type was found in hyrax-associated environments making this a new environmental niche for *C*. *gattii*. We did not find any VGV isolates in our collection of clinical *C*. *gattii* isolates. As Farrer and colleagues conclude, it is possible that this molecular type is strictly environmental and yet to cause clinical infection [22]. Further studies of clinical and environmental *C*. *gattii* isolates from southern Africa should be performed to gain a better understanding of this pathogenic species complex [10].

In conclusion, our study showed that South African clinical *C*. *gattii* isolates, collected through enhanced laboratory-based surveillance from 2005–2013, were highly diverse, the VGIV molecular type dominated, and these isolates also mostly had low fluconazole MIC values. HIV-seropositive patients were more likely to be infected with VGIV isolates. We observed two interesting clusters of closely related VGIV isolates, with one cluster consisting of patients from the Mpumalanga Province in South Africa, suggesting a similar environmental source. Our large study provides a broader baseline for more extensive WGS studies in southern Africa, to enable us to gain a more complete picture of *C*. *gattii*, the lesser known cause of HIV-associated cryptococcal meningitis in this part of the world.

## Supporting information

S1 Fig *Cryptococcus gattii* isolates selected for genotyping from South African laboratory-based surveillance, 2005–2013. Note: NHLS–National Health Laboratory Service, CSF–Cerebrospinal fluid.
(DOCX)

S1 Table GenBank accession numbers for the studied *Cryptococcus gattii* isolates from South Africa, 2005–2013. (DOCX)

S2 Table Nineteen *Cryptococcus gattii* isolates with the VGIV molecular type from the Australian Medical Mycology Culture Collection (WFCC registration number: WM-1205) sequenced in this study. (DOCX)

S3 Table Univariable analysis to determine associations between clinical characteristics and infecting strain molecular type among South African patients infected with *Cryptococcus gattii* (n = 146), 2005–2013. (DOCX)

S4 Table Multivariable logistic regression analysis to determine associations between clinical characteristics and infecting strain molecular type among South African patients infected with *Cryptococcus gattii* (n = 146), 2005–2013. (DOCX)

S5 Table Univariable logistic regression analysis to determine association between infecting strain molecular type and in-hospital outcome among South African patients (n = 142) infected with *Cryptococcus gattii*, 2005–2013. (DOCX)

S6 Table Multivariable logistic regression analysis to determine association between infecting strain molecular type and in-hospital outcome, adjusted for potential confounders, among South African patients (n = 142) infected with *Cryptococcus gattii*, 2005–2013. (DOCX)

S7 Table Characteristics of ten South African patients infected with the *Cryptococcus gattii* VGIV molecular type from the Limpopo and Gauteng Provinces that clustered closely together on WGS analysis as shown in Fig 3A; these isolates were collected during enhanced laboratory-based surveillance for cryptococcosis, 2005–2013. (DOCX)

S8 Table Characteristics of six South African patients infected with the *Cryptococcus gattii* VGIV molecular type from the Mpumalanga Province that clustered closely together on WGS analysis as shown in Fig 3B; these isolates were collected during enhanced laboratory-based surveillance for cryptococcosis, 2005–2013. (DOCX)

S1 Supporting Information Spreadsheet–supplementary information for metadata. (XLSX)

S2 Supporting Information Members of GERMS-SA (2005–2013). (DOCX)
