# Supplementary material for: Molecular type distribution and fluconazole susceptibility of clinical Cryptococcus gattii isolates from South African laboratory-based surveillance, 2005–2013
Source: PLoS Negl Trop Dis. 2022 Jun 29;16(6):e0010448. doi: 10.1371/journal.pntd.0010448 (PMC9242473; doi:10.1371/journal.pntd.0010448)
Supplement: S1 Fig — Note: NHLS–National Health Laboratory Service, CSF–Cerebrospinal fluid. (DOCX) [file pntd.0010448.s001.docx]

South African Laboratory based surveillance from 2005-2013 for cryptococcosis: Routine collection and storage of *Cryptococcus* isolates

1 Jul 2008-31 Dec 2013: Enhanced surveillance sites (29 hospitals in 9 provinces), NHLS labs in KwaZulu-Natal province and pathology laboratories in the private, mining, and military sectors.

**Case definition**

- Positive India ink test on CSF
- Positive cryptococcal antigen test on blood or CSF
- Culture of *Cryptococcus* from any specimen

1 Jan 2005-30 Jun 2008: All South African laboratories

Excluded 363 cases where HIV status was unknown

**Supplementary Figure 1:** *Cryptococcus gattii* isolates selected for genotyping from South African laboratory-based surveillance, 2005-2013.

Note: NHLS – National Health Laboratory Service, CSF – Cerebrospinal fluid

146 cases genotyped

All selected but 10 viable

136 randomly selected

374 HIV seropositive patients

13 HIV seronegative patients

781 viable isolates from 750 cases of *C. gattii*

Excluded *C. neoformans*/recurrent isolates/other *Cryptococcus* species
