# Supplementary material for: Molecular type distribution and fluconazole susceptibility of clinical Cryptococcus gattii isolates from South African laboratory-based surveillance, 2005–2013
Source: PLoS Negl Trop Dis. 2022 Jun 29;16(6):e0010448. doi: 10.1371/journal.pntd.0010448 (PMC9242473; doi:10.1371/journal.pntd.0010448)
Supplement: S1 Table — (DOCX) [file pntd.0010448.s002.docx]

**Supplementary Table 1:** GenBank accession numbers for the studied *Cryptococcus gattii* isolates from South Africa, 2005-2013

| Isolate ID | Record | *CAP59* | *GPD1* | IGS1 | *LAC1* | *PLB1* | *SOD1* | *URA5* |
| --- | --- | --- | --- | --- | --- | --- | --- | --- |
| 2 | 1252 | OL908757 | OM317764 | OL908027 | OL908173 | OL908319 | OL908465 | OL908611 |
| 3 | 6185 | OL908758 | OM317765 | OL908028 | OL908174 | OL908320 | OL908466 | OL908612 |
| 4 | 500 | OL908759 | OM317766 | OL908029 | OL908175 | OL908321 | OL908467 | OL908613 |
| 5 | 2769 | OL908760 | OM317767 | OL908030 | OL908176 | OL908322 | OL908468 | OL908614 |
| 7 | 1031 | OL908761 | OM317768 | OL908031 | OL908177 | OL908323 | OL908469 | OL908615 |
| 8 | 1708 | OL908762 | OM317769 | OL908032 | OL908178 | OL908324 | OL908470 | OL908616 |
| 9 | 1977 | OL908763 | OM317770 | OL908033 | OL908179 | OL908325 | OL908471 | OL908617 |
| 10 | 153 | OL908764 | OM317771 | OL908034 | OL908180 | OL908326 | OL908472 | OL908618 |
| 11 | 954 | OL908765 | OM317772 | OL908035 | OL908181 | OL908327 | OL908473 | OL908619 |
| 12 | 1261 | OL908766 | OM317773 | OL908036 | OL908182 | OL908328 | OL908474 | OL908620 |
| 13 | 236 | OL908767 | OM317774 | OL908037 | OL908183 | OL908329 | OL908475 | OL908621 |
| 14 | 918 | OL908768 | OM317775 | OL908038 | OL908184 | OL908330 | OL908476 | OL908622 |
| 15 | 1365 | OL908769 | OM317776 | OL908039 | OL908185 | OL908331 | OL908477 | OL908623 |
| 16 | 1545 | OL908770 | OM317777 | OL908040 | OL908186 | OL908332 | OL908478 | OL908624 |
| 17 | 273 | OL908771 | OM317778 | OL908041 | OL908187 | OL908333 | OL908479 | OL908625 |
| 18 | 303 | OL908772 | OM317779 | OL908042 | OL908188 | OL908334 | OL908480 | OL908626 |
| 19 | 335 | OL908773 | OM317780 | OL908043 | OL908189 | OL908335 | OL908481 | OL908627 |
| 20 | 1999 | OL908774 | OM317781 | OL908044 | OL908190 | OL908336 | OL908482 | OL908628 |
| 21 | 2532 | OL908775 | OM317782 | OL908045 | OL908191 | OL908337 | OL908483 | OL908629 |
| 22 | 2533 | OL908776 | OM317783 | OL908046 | OL908192 | OL908338 | OL908484 | OL908630 |
| 23 | 2736 | OL908777 | OM317784 | OL908047 | OL908193 | OL908339 | OL908485 | OL908631 |
| 24 | 2899 | OL908778 | OM317785 | OL908048 | OL908194 | OL908340 | OL908486 | OL908632 |
| 25 | 3445 | OL908779 | OM317786 | OL908049 | OL908195 | OL908341 | OL908487 | OL908633 |
| 26 | 3876 | OL908780 | OM317787 | OL908050 | OL908196 | OL908342 | OL908488 | OL908634 |
| 27 | 4387 | OL908781 | OM317788 | OL908051 | OL908197 | OL908343 | OL908489 | OL908635 |
| 28 | 5454 | OL908782 | OM317789 | OL908052 | OL908198 | OL908344 | OL908490 | OL908636 |
| 29 | 5566 | OL908783 | OM317790 | OL908053 | OL908199 | OL908345 | OL908491 | OL908637 |
| 30 | 5714 | OL908784 | OM317791 | OL908054 | OL908200 | OL908346 | OL908492 | OL908638 |
| 31 | 6044 | OL908785 | OM317792 | OL908055 | OL908201 | OL908347 | OL908493 | OL908639 |
| 32 | 6487 | OL908786 | OM317793 | OL908056 | OL908202 | OL908348 | OL908494 | OL908640 |
| 33 | 805 | OL908787 | OM317794 | OL908057 | OL908203 | OL908349 | OL908495 | OL908641 |
| 34 | 1005 | OL908788 | OM317795 | OL908058 | OL908204 | OL908350 | OL908496 | OL908642 |
| 35 | 1124 | OL908789 | OM317796 | OL908059 | OL908205 | OL908351 | OL908497 | OL908643 |
| 36 | 1625 | OL908790 | OM317797 | OL908060 | OL908206 | OL908352 | OL908498 | OL908644 |
| 37 | 3917 | OL908791 | OM317798 | OL908061 | OL908207 | OL908353 | OL908499 | OL908645 |
| 38 | 6516 | OL908792 | OM317799 | OL908062 | OL908208 | OL908354 | OL908500 | OL908646 |
| 39 | 88 | OL908793 | OM317800 | OL908063 | OL908209 | OL908355 | OL908501 | OL908647 |
| 40 | 1163 | OL908794 | OM317801 | OL908064 | OL908210 | OL908356 | OL908502 | OL908648 |
| 41 | 1071 | OL908795 | OM317802 | OL908065 | OL908211 | OL908357 | OL908503 | OL908649 |
| 42 | 1778 | OL908796 | OM317803 | OL908066 | OL908212 | OL908358 | OL908504 | OL908650 |
| 43 | 2399 | OL908797 | OM317804 | OL908067 | OL908213 | OL908359 | OL908505 | OL908651 |
| 44 | 3448 | OL908798 | OM317805 | OL908068 | OL908214 | OL908360 | OL908506 | OL908652 |
| 45 | 3943 | OL908799 | OM317806 | OL908069 | OL908215 | OL908361 | OL908507 | OL908653 |
| 46 | 3598 | OL908800 | OM317807 | OL908070 | OL908216 | OL908362 | OL908508 | OL908654 |
| 47 | 3994 | OL908801 | OM317808 | OL908071 | OL908217 | OL908363 | OL908509 | OL908655 |
| 48 | 3749 | OL908802 | OM317809 | OL908072 | OL908218 | OL908364 | OL908510 | OL908656 |
| 49 | 4051 | OL908803 | OM317810 | OL908073 | OL908219 | OL908365 | OL908511 | OL908657 |
| 50 | 4052 | OL908804 | OM317811 | OL908074 | OL908220 | OL908366 | OL908512 | OL908658 |
| 51 | 4069 | OL908805 | OM317812 | OL908075 | OL908221 | OL908367 | OL908513 | OL908659 |
| 52 | 4102 | OL908806 | OM317813 | OL908076 | OL908222 | OL908368 | OL908514 | OL908660 |
| 53 | 274 | OL908807 | OM317814 | OL908077 | OL908223 | OL908369 | OL908515 | OL908661 |
| 54 | 281 | OL908808 | OM317815 | OL908078 | OL908224 | OL908370 | OL908516 | OL908662 |
| 55 | 326 | OL908809 | OM317816 | OL908079 | OL908225 | OL908371 | OL908517 | OL908663 |
| 56 | 499 | OL908810 | OM317817 | OL908080 | OL908226 | OL908372 | OL908518 | OL908664 |
| 57 | 547 | OL908811 | OM317818 | OL908081 | OL908227 | OL908373 | OL908519 | OL908665 |
| 58 | 917 | OL908812 | OM317819 | OL908082 | OL908228 | OL908374 | OL908520 | OL908666 |
| 59 | 1687 | OL908813 | OM317820 | OL908083 | OL908229 | OL908375 | OL908521 | OL908667 |
| 60 | 1764 | OL908814 | OM317821 | OL908084 | OL908230 | OL908376 | OL908522 | OL908668 |
| 61 | 1846 | OL908815 | OM317822 | OL908085 | OL908231 | OL908377 | OL908523 | OL908669 |
| 62 | 2000 | OL908816 | OM317823 | OL908086 | OL908232 | OL908378 | OL908524 | OL908670 |
| 63 | 2028 | OL908817 | OM317824 | OL908087 | OL908233 | OL908379 | OL908525 | OL908671 |
| 64 | 2174 | OL908818 | OM317825 | OL908088 | OL908234 | OL908380 | OL908526 | OL908672 |
| 65 | 2428 | OL908819 | OM317826 | OL908089 | OL908235 | OL908381 | OL908527 | OL908673 |
| 66 | 2390 | OL908820 | OM317827 | OL908090 | OL908236 | OL908382 | OL908528 | OL908674 |
| 67 | 2476 | OL908821 | OM317828 | OL908091 | OL908237 | OL908383 | OL908529 | OL908675 |
| 68 | 2544 | OL908822 | OM317829 | OL908092 | OL908238 | OL908384 | OL908530 | OL908676 |
| 69 | 786 | OL908823 | OM317830 | OL908093 | OL908239 | OL908385 | OL908531 | OL908677 |
| 70 | 70 | OL908824 | OM317831 | OL908094 | OL908240 | OL908386 | OL908532 | OL908678 |
| 71 | 14 | OL908825 | OM317832 | OL908095 | OL908241 | OL908387 | OL908533 | OL908679 |
| 72 | 607 | OL908826 | OM317833 | OL908096 | OL908242 | OL908388 | OL908534 | OL908680 |
| 73 | 543 | OL908827 | OM317834 | OL908097 | OL908243 | OL908389 | OL908535 | OL908681 |
| 74 | 843 | OL908828 | OM317835 | OL908098 | OL908244 | OL908390 | OL908536 | OL908682 |
| 75 | 980 | OL908829 | OM317836 | OL908099 | OL908245 | OL908391 | OL908537 | OL908683 |
| 76 | 1435 | OL908830 | OM317837 | OL908100 | OL908246 | OL908392 | OL908538 | OL908684 |
| 77 | 376 | OL908831 | OM317838 | OL908101 | OL908247 | OL908393 | OL908539 | OL908685 |
| 78 | 1742 | OL908832 | OM317839 | OL908102 | OL908248 | OL908394 | OL908540 | OL908686 |
| 79 | 1643 | OL908833 | OM317840 | OL908103 | OL908249 | OL908395 | OL908541 | OL908687 |
| 80 | 1787 | OL908834 | OM317841 | OL908104 | OL908250 | OL908396 | OL908542 | OL908688 |
| 81 | 1788 | OL908835 | OM317842 | OL908105 | OL908251 | OL908397 | OL908543 | OL908689 |
| 82 | 1686 | OL908836 | OM317843 | OL908106 | OL908252 | OL908398 | OL908544 | OL908690 |
| 83 | 1698 | OL908837 | OM317844 | OL908107 | OL908253 | OL908399 | OL908545 | OL908691 |
| 84 | 1930 | OL908838 | OM317845 | OL908108 | OL908254 | OL908400 | OL908546 | OL908692 |
| 85 | 2085 | OL908839 | OM317846 | OL908109 | OL908255 | OL908401 | OL908547 | OL908693 |
| 86 | 252 | OL908840 | OM317847 | OL908110 | OL908256 | OL908402 | OL908548 | OL908694 |
| 87 | 455 | OL908841 | OM317848 | OL908111 | OL908257 | OL908403 | OL908549 | OL908695 |
| 88 | 841 | OL908842 | OM317849 | OL908112 | OL908258 | OL908404 | OL908550 | OL908696 |
| 89 | 848 | OL908843 | OM317850 | OL908113 | OL908259 | OL908405 | OL908551 | OL908697 |
| 90 | 940 | OL908844 | OM317851 | OL908114 | OL908260 | OL908406 | OL908552 | OL908698 |
| 91 | 924 | OL908845 | OM317852 | OL908115 | OL908261 | OL908407 | OL908553 | OL908699 |
| 92 | 976 | OL908846 | OM317853 | OL908116 | OL908262 | OL908408 | OL908554 | OL908700 |
| 93 | 1037 | OL908847 | OM317854 | OL908117 | OL908263 | OL908409 | OL908555 | OL908701 |
| 94 | 1087 | OL908848 | OM317855 | OL908118 | OL908264 | OL908410 | OL908556 | OL908702 |
| 95 | 1150 | OL908849 | OM317856 | OL908119 | OL908265 | OL908411 | OL908557 | OL908703 |
| 96 | 1280 | OL908850 | OM317857 | OL908120 | OL908266 | OL908412 | OL908558 | OL908704 |
| 97 | 1404 | OL908851 | OM317858 | OL908121 | OL908267 | OL908413 | OL908559 | OL908705 |
| 98 | 1428 | OL908852 | OM317859 | OL908122 | OL908268 | OL908414 | OL908560 | OL908706 |
| 99 | 1723 | OL908853 | OM317860 | OL908123 | OL908269 | OL908415 | OL908561 | OL908707 |
| 100 | 1840 | OL908854 | OM317861 | OL908124 | OL908270 | OL908416 | OL908562 | OL908708 |
| 101 | 1866 | OL908855 | OM317862 | OL908125 | OL908271 | OL908417 | OL908563 | OL908709 |
| 102 | 22 | OL908856 | OM317863 | OL908126 | OL908272 | OL908418 | OL908564 | OL908710 |
| 103 | 339 | OL908857 | OM317864 | OL908127 | OL908273 | OL908419 | OL908565 | OL908711 |
| 104 | 348 | OL908858 | OM317865 | OL908128 | OL908274 | OL908420 | OL908566 | OL908712 |
| 105 | 386 | OL908859 | OM317866 | OL908129 | OL908275 | OL908421 | OL908567 | OL908713 |
| 106 | 465 | OL908860 | OM317867 | OL908130 | OL908276 | OL908422 | OL908568 | OL908714 |
| 107 | 561 | OL908861 | OM317868 | OL908131 | OL908277 | OL908423 | OL908569 | OL908715 |
| 108 | 1127 | OL908862 | OM317869 | OL908132 | OL908278 | OL908424 | OL908570 | OL908716 |
| 109 | 1385 | OL908863 | OM317870 | OL908133 | OL908279 | OL908425 | OL908571 | OL908717 |
| 110 | 1783 | OL908864 | OM317871 | OL908134 | OL908280 | OL908426 | OL908572 | OL908718 |
| 111 | 2025 | OL908865 | OM317872 | OL908135 | OL908281 | OL908427 | OL908573 | OL908719 |
| 112 | 2026 | OL908866 | OM317873 | OL908136 | OL908282 | OL908428 | OL908574 | OL908720 |
| 113 | 2133 | OL908867 | OM317874 | OL908137 | OL908283 | OL908429 | OL908575 | OL908721 |
| 114 | 2172 | OL908868 | OM317875 | OL908138 | OL908284 | OL908430 | OL908576 | OL908722 |
| 115 | 2173 | OL908869 | OM317876 | OL908139 | OL908285 | OL908431 | OL908577 | OL908723 |
| 116 | 2259 | OL908870 | OM317877 | OL908140 | OL908286 | OL908432 | OL908578 | OL908724 |
| 117 | 2217 | OL908871 | OM317878 | OL908141 | OL908287 | OL908433 | OL908579 | OL908725 |
| 119 | 2569 | OL908872 | OM317879 | OL908142 | OL908288 | OL908434 | OL908580 | OL908726 |
| 120 | 2414 | OL908873 | OM317880 | OL908143 | OL908289 | OL908435 | OL908581 | OL908727 |
| 121 | 2612 | OL908874 | OM317881 | OL908144 | OL908290 | OL908436 | OL908582 | OL908728 |
| 122 | 2843 | OL908875 | OM317882 | OL908145 | OL908291 | OL908437 | OL908583 | OL908729 |
| 123 | 2970 | OL908876 | OM317883 | OL908146 | OL908292 | OL908438 | OL908584 | OL908730 |
| 124 | 3058 | OL908877 | OM317884 | OL908147 | OL908293 | OL908439 | OL908585 | OL908731 |
| 125 | 3096 | OL908878 | OM317885 | OL908148 | OL908294 | OL908440 | OL908586 | OL908732 |
| 126 | 3332 | OL908879 | OM317886 | OL908149 | OL908295 | OL908441 | OL908587 | OL908733 |
| 127 | 3470 | OL908880 | OM317887 | OL908150 | OL908296 | OL908442 | OL908588 | OL908734 |
| 128 | 3772 | OL908881 | OM317888 | OL908151 | OL908297 | OL908443 | OL908589 | OL908735 |
| 129 | 3796 | OL908882 | OM317889 | OL908152 | OL908298 | OL908444 | OL908590 | OL908736 |
| 130 | 3882 | OL908883 | OM317890 | OL908153 | OL908299 | OL908445 | OL908591 | OL908737 |
| 131 | 4145 | OL908884 | OM317891 | OL908154 | OL908300 | OL908446 | OL908592 | OL908738 |
| 133 | 4274 | OL908885 | OM317892 | OL908155 | OL908301 | OL908447 | OL908593 | OL908739 |
| 134 | 4009 | OL908886 | OM317893 | OL908156 | OL908302 | OL908448 | OL908594 | OL908740 |
| 135 | 75 | OL908887 | OM317894 | OL908157 | OL908303 | OL908449 | OL908595 | OL908741 |
| 136 | 2083 | OL908888 | OM317895 | OL908158 | OL908304 | OL908450 | OL908596 | OL908742 |
| 137 | 772 | OL908889 | OM317896 | OL908159 | OL908305 | OL908451 | OL908597 | OL908743 |
| 138 | 3360 | OL908890 | OM317897 | OL908160 | OL908306 | OL908452 | OL908598 | OL908744 |
| 139 | 2060 | OL908891 | OM317898 | OL908161 | OL908307 | OL908453 | OL908599 | OL908745 |
| 140 | 1722 | OL908892 | OM317899 | OL908162 | OL908308 | OL908454 | OL908600 | OL908746 |
| 141 | 2505 | OL908893 | OM317900 | OL908163 | OL908309 | OL908455 | OL908601 | OL908747 |
| 142 | 2530 | OL908894 | OM317901 | OL908164 | OL908310 | OL908456 | OL908602 | OL908748 |
| 143 | 3954 | OL908895 | OM317902 | OL908165 | OL908311 | OL908457 | OL908603 | OL908749 |
| 144 | 3995 | OL908896 | OM317903 | OL908166 | OL908312 | OL908458 | OL908604 | OL908750 |
| 145 | 2854 | OL908897 | OM317904 | OL908167 | OL908313 | OL908459 | OL908605 | OL908751 |
| 146 | 1751 | OL908898 | OM317905 | OL908168 | OL908314 | OL908460 | OL908606 | OL908752 |
| 147 | 841 | OL908899 | OM317906 | OL908169 | OL908315 | OL908461 | OL908607 | OL908753 |
| 148 | 2153 | OL908900 | OM317907 | OL908170 | OL908316 | OL908462 | OL908608 | OL908754 |
| 149 | 3061 | OL908901 | OM317908 | OL908171 | OL908317 | OL908463 | OL908609 | OL908755 |
| 150 | 3234 | OL908902 | OM317909 | OL908172 | OL908318 | OL908464 | OL908610 | OL908756 |
