# Supplementary material for: Molecular type distribution and fluconazole susceptibility of clinical Cryptococcus gattii isolates from South African laboratory-based surveillance, 2005–2013
Source: PLoS Negl Trop Dis. 2022 Jun 29;16(6):e0010448. doi: 10.1371/journal.pntd.0010448 (PMC9242473; doi:10.1371/journal.pntd.0010448)
Supplement: S2 Table — (DOCX) [file pntd.0010448.s003.docx]

**Supplementary Table 2:** Nineteen *Cryptococcus gattii* isolates with the VGIV molecular type from the Australian Medical Mycology Culture Collection (WFCC registration number: WM-1205) sequenced in this study

| **Strain Number** | **Other number/s** | **Country** | **Source** | **Year of isolation** |
| --- | --- | --- | --- | --- |
| WM 04.20 | CBS11254 M27055 M25229 | South Africa | Clinical | 1996 |
| WM 05.373 | H0058-I-1875 | Colombia | Eucalyptus tree | 2003 |
| WM 05.374 | H0058-I-1876 | Colombia | Eucalyptus tree | 2003 |
| WM 05.376 | H0058-I-1686 | Colombia | Almond tree | 2003 |
| WM 05.378 | H0058-I-1688 | Colombia | Almond tree | 2003 |
| WM 08.314 | 16-1664  W552  M20724 Mbatha | South Africa | Unknown | Unknown |
| WM 12.43 | 01-201083 | Australia | Clinical (Cerebrospinal fluid) | 2001 |
| WM 12.66 | AMMRL 30.135 | Australia | Clinical (Cerebrospinal fluid) | 2005 |
| WM 14.321 | 106.97 | Unknown | Unknown | Unknown |
| WM 1434 | UON 11135 | South Africa | Clinical (Cerebrospinal fluid) | 1997 |
| WM 2363 | B5742 CBS11248  MP-8/92 MCL250025 | India | Clinical (Cerebrospinal fluid) | 1997 |
| WM 2567 | M27042  X242 | South Africa | Clinical | 1996 |
| WM 2568 | M27043  X303 | South Africa | Clinical | 1996 |
| WM 2570 | M27046  P2244 | South Africa | Clinical | 1996 |
| WM 2579 | M27056  P2238 | South Africa | Clinical | 1996 |
| WM 2604 | M31499  4357 | South Africa | Clinical | 1996 |
| WM 2670 | MJ-33 | South Africa | Clinical | 1996 |
| WM 2876 | V00869 | South Africa | Clinical | 1996 |
| WM2364 | B5748 CBS11249 P496  MCL250024 | India | Clinical | 1997 |
