# Supplementary material for: Molecular type distribution and fluconazole susceptibility of clinical Cryptococcus gattii isolates from South African laboratory-based surveillance, 2005–2013
Source: PLoS Negl Trop Dis. 2022 Jun 29;16(6):e0010448. doi: 10.1371/journal.pntd.0010448 (PMC9242473; doi:10.1371/journal.pntd.0010448)
Supplement: S3 Table — (DOCX) [file pntd.0010448.s004.docx]

**Supplementary Table 3:** Univariable analysis to determine associations between clinical characteristics and infecting strain molecular type among South African patients infected with *Cryptococcus gattii* (n=146), 2005-2013

| **Exposure variables** | **VGIV** | **Non-VGIV** | **Univariable analysis** | |
| --- | --- | --- | --- | --- |
|  | **N = 101** | **N = 45** |  | |
|  | **n/N (%)** | **n/N (%)** | **OR (95% CI)** | **p-value** |
| **Sex** |  |  |  |  |
| Male | 56/85 (66) | 29/85 (34) | 0.69 (0.33-1.42) | 0.31 |
| Female | 45/61 (74) | 16/61 (26) | Reference |  |
| **Age (years)** |  |  |  |  |
| <25 | 11/17 (65) | 6/17 (35) | Reference |  |
| 25-34 | 36/50 (72) | 14/50 (28) | 1.40 (0.44-4.52) | 0.57 |
| 35-44 | 41/55 (75) | 14/55 (25) | 1.60 (0.50-5.12) | 0.43 |
| >45 | 13/24 (54) | 11/24 (46) | 0.64 (0.18-2.31) | 0.50 |
| **Year of diagnosis** |  |  |  |  |
| 2005 | 2/4 (50) | 2/4 (50) | Reference |  |
| 2006 | 12/19 (63) | 7/19 (37) | 1.71 (0.20-15.02) | 0.63 |
| 2007 | 9/9 (100) | 0/9 (0) |  |  |
| 2008 | 12/18 (67) | 6/18 (33) | 2.00 (0.22-17.89) | 0.54 |
| 2009 | 16/20 (80) | 4/20 (20) | 4.00 (0.42-37.78) | 0.23 |
| 2010 | 12/21 (57) | 9/21 (43) | 1.33 (0.16-11.36) | 0.79 |
| 2011 | 16/21 (76) | 5/21 (24) | 3.20 (0.35-28.94) | 0.30 |
| 2012 | 7/11 (64) | 4/11 (36) | 1.75 (0.17-17.69) | 0.64 |
| 2013 | 15/23 (65) | 8/23 (35) | 1.88 (0.22-15.93) | 0.57 |
| **Geographical region^*^** |  |  |  |  |
| Temperate | 79/117 (68) | 38/117 (32) | 0.66 (0.26-1.68) | 0.39 |
| Arid | 22/29 (76) | 7/29 (24) | Reference |  |
| **Specimen type** |  |  |  |  |
| Cerebrospinal fluid (CSF) | 95/137 (69) | 42/137 (31) | 0.45 (0.05-3.99) | 0.48 |
| Blood | 5/6 (83) | 1/6 (17) | Reference |  |
| Missing data (n) | 1 | 2 |  |  |
| **HIV infection status** |  |  |  |  |
| Positive | 99/136 (73) | 37/136 (27) | 10.70 (2.17-52.74) | 0.004 |
| Negative | 2/10 (20) | 8/10 (80) | Reference |  |
| **CD4+ T-cell count at diagnosis (cells/µl)** |  |  |  |  |
| <=50 | 37/46 (80) | 9/46 (20) | 1.67 (0.63-4.42) | 0.30 |
| >50 | 32/45 (71) | 13/45 (29) | Reference |  |
| Missing data (n) | 32 | 23 |  |  |
| **Antiretroviral treatment** |  |  |  |  |
| Yes | 38/55 (69) | 17/55 (31) | 0.80 (0.37-1.72) | 0.57 |
| No | 56/76 (74) | 20/76 (26) | Reference |  |
| Missing data (n) | 7 | 8 |  |  |
| **Mental status at diagnosis^*^** |  |  |  |  |
| Alert | 69/97 (71) | 28/97 (29) | 1.33 (0.61-2.91) | 0.48 |
| Not alert | 26/40 (65) | 14/40 (35) | Reference |  |
| Missing data (n) | 6 | 3 |  |  |
| **Current antifungal treatment** |  |  |  |  |
| Fluconazole alone | 19/30 (63) | 11/30 (37) | Reference |  |
| Fluconazole and  amphotericin B | 50/70 (71) | 20/70 (29) | 1.45 (0.59-3.58) | 0.42 |
| Missing data (n) | 32 | 14 |  |  |
| **Current TB treatment** |  |  |  |  |
| Yes | 28/41 (68) | 13/41 (32) | 0.88 (0.39-1.95) | 0.74 |
| No | 64/90 (71) | 26/90 (29) | Reference |  |
| Missing data (n) | 9 | 6 |  |  |

*Geographical region was categorised as mostly temperate (Gauteng, Mpumalanga, KwaZulu-Natal and Western Cape provinces) or mostly arid (Northern Cape, Free State, Limpopo, North West and Eastern Cape provinces). *Mental status was categorised as “Alert” (Glasgow Coma Scale [GCS] score of 15) or “Not alert” (GCS score of <15 or recorded to be disorientated, stuporose or comatose).
