# Supplementary material for: Molecular type distribution and fluconazole susceptibility of clinical Cryptococcus gattii isolates from South African laboratory-based surveillance, 2005–2013
Source: PLoS Negl Trop Dis. 2022 Jun 29;16(6):e0010448. doi: 10.1371/journal.pntd.0010448 (PMC9242473; doi:10.1371/journal.pntd.0010448)
Supplement: S4 Table — (DOCX) [file pntd.0010448.s005.docx]

**Supplementary Table 4:** Multivariable logistic regression analysis to determine associations between clinical characteristics and infecting strain molecular type among South African patients infected with *Cryptococcus gattii* (n=146), 2005-2013

| **Exposure variables** | **VGIV** | **Non-VGIV** | **Multivariable analysis** | |
| --- | --- | --- | --- | --- |
|  | **N = 101** | **N = 45** |  | |
|  | **n/N (%)** | **n/N (%)** | **aOR (95% CI)** | **p-value** |
| **Geographical region** |  |  |  |  |
| Temperate | 79/117 (68) | 38/117 (32) | 0.57 (0.21-1.58) | 0.28 |
| Arid | 22/29 (76) | 7/29 (24) | Reference |  |
| **HIV infection status** |  |  |  |  |
| Positive | 99/136 (73) | 37/136 (27) | 10.34 (1.93-55.31) | 0.006 |
| Negative | 2/10 (20) | 8/10 (80) | Reference |  |
| **Sex** |  |  |  |  |
| Male | 56/85 (66) | 29/85 (34) | 0.57 (0.25-1.28) | 0.18 |
| Female | 45/61 (74) | 16/61 (26) | Reference |  |
| **Age (years)** |  |  |  |  |
| <25 | 11/17 (65) | 6/17 (35) | Reference |  |
| 25-34 | 36/50 (72) | 14/50 (28) | 0.77 (0.20-3.03) | 0.71 |
| 35-44 | 41/55 (75) | 14/55 (25) | 1.00 (0.25-3.93) | 1.0 |
| >45 | 13/24 (54) | 11/24 (46) | 0.42 (0.10-1.84) | 0.25 |
