# Supplementary material for: Molecular type distribution and fluconazole susceptibility of clinical Cryptococcus gattii isolates from South African laboratory-based surveillance, 2005–2013
Source: PLoS Negl Trop Dis. 2022 Jun 29;16(6):e0010448. doi: 10.1371/journal.pntd.0010448 (PMC9242473; doi:10.1371/journal.pntd.0010448)
Supplement: S5 Table — (DOCX) [file pntd.0010448.s006.docx]

**Supplementary Table 5:** Univariable logistic regression analysis to determine association between infecting strain molecular type and in-hospital outcome among South African patients (n=142) infected with *Cryptococcus gattii*, 2005-2013

| **Exposure variables** | **Died** | **Survived** | **Univariable analysis** | |
| --- | --- | --- | --- | --- |
|  | **N = 40** | **N = 102** |  | |
|  | **n/N (%)** | **n/N (%)** | **OR (95% CI)** | **p-value** |
| **Molecular type** |  |  |  |  |
| VGIV | 29/98 (30) | 69/98 (70) | 1.26 (0.56-2.83) | 0.57 |
| Non-VGIV | 11/44 (25) | 33/44 (75) | Reference |  |
| **Sex** |  |  |  |  |
| Male | 18/83 (22) | 65/83 (78) | 0.47 (0.22-0.99) | 0.05 |
| Female | 22/59 (37) | 37/59 (63) | Reference |  |
| **Age (years)** |  |  |  |  |
| <25 | 3/17 (18) | 14/17 (82) | Reference |  |
| 25-34 | 15/48 (31) | 33/48 (69) | 2.10 (0.52-8.41) | 0.30 |
| 35-44 | 15/53 (28) | 38/53 (72) | 1.80 (0.45-7.20) | 0.41 |
| ≥45 | 7/24 (29) | 17/24 (71) | 1.97 (0.43-9.11) | 0.39 |
| **Year of diagnosis** |  |  |  |  |
| 2005 | 1/4 (25) | 3/4 (75) | Reference |  |
| 2006 | 4/19 (21) | 15/19 (79) | 0.75 (0.06-9.48) | 0.83 |
| 2007 | 1/9 (11) | 8/9 (89) | 0.30 (0.01-6.80) | 0.45 |
| 2008 | 5/18 (28) | 13/18 (72) | 1.08  (0.09-13.12) | 0.96 |
| 2009 | 3/18 (17) | 15/18 (83) | 0.52 (0.04-7.04) | 0.62 |
| 2010 | 4/21 (19) | 17/21 (81) | 0.68 (0.05-8.51) | 0.77 |
| 2011 | 6/21 (29) | 15/21 (71) | 1.07  (0.09-12.74) | 0.96 |
| 2012 | 5/9 (56) | 4/9 (44) | 3.71  (0.27-51.52) | 0.33 |
| 2013 | 11/23 (48) | 12/23 (52) | 2.60  (0.23-29.22) | 0.44 |
| **Hospital** |  |  |  |  |
| Academic | 18/64 (28) | 46/64 (72) | 1.01 (0.48-2.11) | 0.98 |
| Regional | 22/78 (28) | 56/78 (72) | Reference |  |
| **Specimen type** |  |  |  |  |
| Cerebrospinal fluid (CSF) | 37/134 (28) | 97/134 (72) | 0.80 (0.14-4.58) | 0.80 |
| Blood | 2/6 (33) | 4/6 (67) | Reference |  |
| Missing data (n) | 1 | 1 |  |  |
| **CD4+ T-cell count at diagnosis (cells/µl)** |  |  |  |  |
| ≤50 | 14/45 (31) | 31/45 (69) | 1.36 (0.54-3.46) | 0.52 |
| >50 | 11/45 (24) | 34/45 (76) | Reference |  |
| Missing data (n) | 15 | 37 |  |  |
| **Antiretroviral treatment** |  |  |  |  |
| Yes | 13/54 (24) | 41/54 (76) | 0.82 (0.36-1.84) | 0.63 |
| No | 21/74 (28) | 53/74 (72) | Reference |  |
| Missing data (n) | 6 | 8 |  |  |
| **Mental status at diagnosis*** |  |  |  |  |
| Alert | 15/94 (16) | 79/94 (84) | 0.18 (0.08-0.41) | 0.001 |
| Not alert | 20/39 (51) | 19/39 (49) | Reference |  |
| Missing data (n) | 5 | 4 |  |  |
| **Current antifungal treatment** |  |  |  |  |
| Fluconazole alone | 7/29 (24) | 22/29 (76) | Reference |  |
| Fluconazole and  amphotericin B | 15/69 (22) | 54/69 (78) | 0.89 (0.32-2.49) | 0.82 |
| Missing data (n) | 18 | 26 |  |  |
| **Current tuberculosis treatment** |  |  |  |  |
| Yes | 18/41 (44) | 23/41 (56) | 3.79 (1.64-8.75) | 0.002 |
| No | 15/86 (17) | 71/86 (83) | Reference |  |
| Missing data (n) | 7 | 8 |  |  |

*Mental status was categorised as “Alert” (Glasgow Coma Scale [GCS] score of 15) or “Not alert” (GCS score of <15 or recorded to be disorientated, stuporose or comatose).
