# Supplementary material for: Molecular type distribution and fluconazole susceptibility of clinical Cryptococcus gattii isolates from South African laboratory-based surveillance, 2005–2013
Source: PLoS Negl Trop Dis. 2022 Jun 29;16(6):e0010448. doi: 10.1371/journal.pntd.0010448 (PMC9242473; doi:10.1371/journal.pntd.0010448)
Supplement: S6 Table — (DOCX) [file pntd.0010448.s007.docx]

**Supplementary Table 6:** Multivariable logistic regression analysis to determine association between infecting strain molecular type and in-hospital outcome, adjusted for potential confounders, among South African patients (n=142) infected with *Cryptococcus gattii*, 2005-2013

| **Exposure variables** | **Died** | **Survived** | **Multivariable analysis** | |
| --- | --- | --- | --- | --- |
|  | **N = 40** | **N = 102** |  | |
|  | **n/N (%)** | **n/N (%)** | **aOR (95% CI)** | **p-value** |
| **Molecular type** |  |  |  |  |
| VGIV | 29/98 (30) | 69/98 (70) | 0.57 (0.07-4.43) | 0.59 |
| Non-VGIV | 11/44 (25) | 33/44 (75) | reference |  |
| **Sex** |  |  |  |  |
| Male | 18/83 (22) | 65/83 (78) | 0.72 (0.12-4.27) | 0.72 |
| Female | 22/59 (37) | 37/59 (63) | reference |  |
| **Age (years)** |  |  |  |  |
| <25 | 3/17 (18) | 14/17 (82) | reference |  |
| 25-34 | 15/48 (31) | 33/48 (69) | 0.48 (0.02-9.71) | 0.64 |
| 35-44 | 15/53 (28) | 38/53 (72) | 0.54 (0.03-9.39) | 0.67 |
| ≥45 | 7/24 (29) | 17/24 (71) | 0.61 (0.02-15.40) | 0.76 |
| **CD4+ T-cell count at diagnosis (cells/µl)** |  |  |  |  |
| ≤50 | 14/45 (31) | 31/45 (69) | 2.26 (0.35-14.43) | 0.39 |
| >50 | 11/45 (24) | 34/45 (76) | reference |  |
| Missing data (n) | 15 | 37 |  |  |
| **Antiretroviral treatment** |  |  |  |  |
| Yes | 13/54 (24) | 41/54 (76) | 0.64 (0.11-3.59) | 0.61 |
| No | 21/74 (28) | 53/74 (72) | reference |  |
| Missing data (n) | 6 | 8 |  |  |
| **Mental status at diagnosis*** |  |  |  |  |
| Alert | 15/94 (16) | 79/94 (84) | 0.20 (0.03-1.27) | 0.09 |
| Not alert | 20/39 (51) | 19/39 (49) | reference |  |
| Missing data (n) | 5 | 4 |  |  |
| **Current antifungal treatment** |  |  |  |  |
| Fluconazole alone | 7/29 (24) | 22/29 (76) | reference |  |
| Fluconazole and amphotericin B | 15/69 (22) | 54/69 (78) | 1.65 (0.13-20.66) | 0.70 |
| Missing data (n) | 18 | 26 |  |  |

*Mental status was categorised as “Alert” (Glasgow Coma Scale [GCS] score of 15) or “Not alert” (GCS score of <15 or recorded to be disorientated, stuporose or comatose).
