# Supplementary material for: Molecular type distribution and fluconazole susceptibility of clinical Cryptococcus gattii isolates from South African laboratory-based surveillance, 2005–2013
Source: PLoS Negl Trop Dis. 2022 Jun 29;16(6):e0010448. doi: 10.1371/journal.pntd.0010448 (PMC9242473; doi:10.1371/journal.pntd.0010448)
Supplement: S7 Table — (DOCX) [file pntd.0010448.s008.docx]

**Supplementary Table 7**: Characteristics of ten South African patients infected with the *Cryptococcus gattii* VGIV molecular type from the Limpopo and Gauteng Provinces that clustered closely together on WGS analysis as shown in Fig 3A; these isolates were collected during enhanced laboratory-based surveillance for cryptococcosis, 2005-2013

| **Isolate number** | **Home town/city** | **Province** | **Specimen collection date** | **Syndrome** | **Facility name** |
| --- | --- | --- | --- | --- | --- |
| 2428 | Polokwane | Limpopo | 18/11/2009 | Meningitis | Mankweng |
| 2612 | Pretoria | Gauteng | 14/4/2013 | Meningitis | Dr George Mukhari |
| 2133 | Northcliff | Gauteng | 23/1/2013 | Meningitis | Helen Joseph |
| 153 | Polokwane | Limpopo | 23/1/2009 | Meningitis and pneumonia | Polokwane |
| 918 | Polokwane | Limpopo | 20/5/2010 | Meningitis | Polokwane |
| 4145 | Tzaneen | Limpopo | 2/11/2013 | Meningitis | Mankweng |
| 2476 | Polokwane | Limpopo | 14/12/2009 | Meningitis | Mankweng |
| 1163 | Polokwane | Limpopo | 20/2/2008 | Meningitis | Mankweng |
| 6487 | Eldorado Park | Gauteng | 27/12/2006 | Meningitis | Chris Hani Baragwanath |
| 772 | Polokwane | Limpopo | 5/8/2011 | Meningitis | Polokwane |
