# Supplementary material for: Molecular type distribution and fluconazole susceptibility of clinical Cryptococcus gattii isolates from South African laboratory-based surveillance, 2005–2013
Source: PLoS Negl Trop Dis. 2022 Jun 29;16(6):e0010448. doi: 10.1371/journal.pntd.0010448 (PMC9242473; doi:10.1371/journal.pntd.0010448)
Supplement: S8 Table — (DOCX) [file pntd.0010448.s009.docx]

**Supplementary Table 8**: Characteristics of six South African patients infected with the *Cryptococcus gattii* VGIV molecular type from the Mpumalanga Province that clustered closely together on WGS analysis as shown in Fig 3B; these isolates were collected during enhanced laboratory-based surveillance for cryptococcosis, 2005-2013

| **Isolate number** | **Home town/city** | **Province** | **Specimen collection date** | **Syndrome** | **Facility name** |
| --- | --- | --- | --- | --- | --- |
| 70 | Nelspruit | Mpumalanga | 14/1/2010 | Meningitis | Themba |
| 2153 | Kabokweni | Mpumalanga | 27/4/2008 | Meningitis | Themba |
| 976 | Barberton | Mpumalanga | 14/7/2011 | Meningitis | Rob Ferreira |
| 1261 | Kabokweni | Mpumalanga | 15/6/2009 | Meningitis | Themba |
| 1999 | Kabokweni | Mpumalanga | 5/5/2006 | Meningitis | Themba |
| 2085 | White River | Mpumalanga | 16/12/2010 | Meningitis | Themba |
