# Supplementary material for: Molecular type distribution and fluconazole susceptibility of clinical Cryptococcus gattii isolates from South African laboratory-based surveillance, 2005–2013
Source: PLoS Negl Trop Dis. 2022 Jun 29;16(6):e0010448. doi: 10.1371/journal.pntd.0010448 (PMC9242473; doi:10.1371/journal.pntd.0010448)
Supplement: S2 Supporting Information — (DOCX) [file pntd.0010448.s011.docx]

**Members of GERMS-SA (2005-2013)**

Pieter Ekermans, Patricia Hanise, Carel Haummann, Dania Perez, Sandeep Vasaikar (Eastern Cape); Gene Elliott, Dominique Goedhals, Ute Halbauer, Anwar Hoosen, Madeleine Pieters, Justyna Wojno (Free State); Theunis Avenant, Kamaldeen Baba, Norma Bosman, Vindana Chibabhai, Jeane Cloete, Nicolette du Plessis, Adriano Duse, Charles Feldman, Alan Karstaedt, Ranmini Kularatne, Ruth Lekalakala, Kathy Lindeque, Warren Lowman, Motlatji Maloba, Caroline Maluleka, Moamekgethi Moshe, Trusha Nana, Maphoshane Nchabeleng, Gary Reubenson, Sharona Seetharam, Sheeba Varughese, Charl Verwey, Jeannette Wadula (Gauteng); Moherndran Archary, Prathna Bhola, Asmeeta Burra, Yacoob Coovadia, Halima Dawood, Khatija Dawood, Sumayya Haffejee, Prasha Mahabeer, Koleka Mlisana, Fathima Naby, Romola Naidoo, Praksha Ramjathan, Lisha Sookan, Khine Swe Swe Han (KwaZulu Natal); Andries Dreyer, Ken Hamese, Phaswe Maredi (Limpopo); Greta Hoyland, Jacob Lebudi, Barry Spies (Mpumalanga); Riezaah Abrahams, Dhamiran Naidoo, Eunice Weenink (Northern Cape); Andrew Rampe, Eduard Silberbauer, Lino Sono, Ebrahim Variava (North West); Colleen Bamford, Louise Cooke, Brian Eley, Heather Finlayson, Rena Hoffmann, Preneshni Naicker, Mark Nicol, James Nuttal, Heidi Orth, Helena Rabie, Catherine Samuel, Andrew Whitelaw (Western Cape); Maria Botha, Adrian Brink, Mark Cruz de Silva, Chanelle Moore, Xoliswa Poswa, Peter Smith, Charlotte Sriruttan, Inge Zietsman (AMPATH); Chetna Govind, Krishnee Moodley, Keshree Pillay, Juanita Smit (LANCET); Marthinus Senekal (PathCare); Stephanie Schrag, Jennifer Verani, Cynthia Whitney (CDC); Keith Klugman (Emory); Penny Crowther-Gibson, Cheryl Cohen, Linda de Gouveia, Linda Erasmus, Melony Fortuin-de Smidt, Nelesh Govender, Nevashan Govender, Nazir Ismail, Karen Keddy, Sarona Lengana, Sonwabo Lindani, Susan Meiring, Cecilia Miller, Ruth Mpembe, Nireshni Naidoo, Ananta Nanoo, Tsakane Nkuna, Olga Perovic, Vanessa Quan, Mmakgomo Rakhudu, Languta Sibiya, Marshagne Smith, Arvinda Sooka, Anne von Gottberg, Claire von Mollendorf, Bulelwa Zigana (NICD).
